# A Honduran Prevalence Study on Soil-Transmitted Helminths Highlights Serological Antibodies to *Tm*-WAP49 as a Diagnostic Marker for Exposure to Human Trichuriasis

**DOI:** 10.4269/ajtmh.24-0514

**Published:** 2025-02-11

**Authors:** Neima Briggs, Leroy Versteeg, Rojelio Mejia, Jeroen Pollet, Maria Jose Villar, Bin Zhan, Graeme Segal, Stephanie Novak, Patricia Lenihan, Paul Musgrave, Viviana Ellis, Carol Florencia Coello, K. Jagannadha Sastry, Joe Craft, Peter J. Hotez, Maria Elena Bottazzi

**Affiliations:** ^1^ Department of Internal Medicine (Infectious Diseases), Yale University School of Medicine, New Haven, Connecticut;; ^2^ Department of Immunobiology, Yale University School of Medicine, New Haven, Connecticut;; ^3^ Texas Children’s Hospital Center for Vaccine Development, Department of Pediatric Tropical Medicine, National School of Tropical Medicine, Baylor College of Medicine, Houston, Texas;; ^4^ McGovern Medical School at the University of Texas Health Science Center at Houston, Houston, Texas;; ^5^ Santa Ana Clinic, Houston Shoulder to Shoulder Foundation, Houston, Texas;; ^6^ Department of Thoracic Head and Neck Medical Oncology, The University of Texas MD Anderson Cancer Center, Houston, Texas;; ^7^ Department of Internal Medicine (Rheumatology, Allergy and Immunology), Yale University School of Medicine, New Haven, Connecticut;; ^8^ Department of Molecular Virology and Microbiology, Baylor College of Medicine, Houston, Texas;; ^9^ Department of Biology, Baylor University, Waco, Texas;; ^10^ James A. Baker III Institute for Public Policy, Rice University, Houston, Texas;; ^11^ Hagler Institute for Advanced Study, Texas A&M University, College Station, Texas

## Abstract

Soil-transmitted helminth (STH) infections rank among the most prevalent communicable diseases of humans, yet detection of these parasites is mostly restricted to identifying active infection through fecal examinations. Currently, there are no commercial diagnostic tools to identify a prior whipworm or hookworm exposure, and the few serological assays for roundworm infection have not been well validated for crossreactivity or infections in humans. Such diagnostic restrictions limit the range of scientific and clinical questions that surround STH exposures and their implicated relationship to chronic diseases, such as autoimmunity, allergy, and cancer. The goal of this investigation was to evaluate the diagnostic potential of 13 STH recombinant proteins. As there are no gold standard tests to verify positive STH antisera, we used sera from active STH-infected individuals in Honduras (measured by quantitative real-time polymerase chain reaction of helminth DNA in stool) and compared antibody recognition by both ELISA and western blot with nonendemic control sera from age-matched individuals in the United States split into screening and validation cohorts. One recombinant protein, r*Tm*-WAP49, shows potential as a whipworm diagnostic tool by receiver-operator characteristic analysis (area under the curve = 0.997, *P* <0.001) and indirect ELISA with sensitivity of 100% and specificity of 91% as defined by mean plus two SDs from the nonendemic screening cohort. We found discrepancies in serological recognition of previously tested STH antigens, highlighting the need to consider different technologies before down selection of a promising diagnostic candidate and screen multiple endemic populations before widely accepting an STH serological assay.

## INTRODUCTION

Nearly 1.5 billion people, a quarter of the world’s population, are infected with a soil-transmitted helminth (STH).^1^ Consisting of hookworm (predominantly *Necator americanus* but also* Ancylostoma duodenale*, and *Ancylostoma ceylanicum*), whipworm (*Trichuris trichiura*), roundworm (*Ascaris lumbricoides*), and threadworm (*Strongyloides stercoralis*), these neglected tropical diseases are endemic to most of the world’s temperate and tropical regions.

Through multiple mechanisms, STH infections are known to reinforce the cycle of poverty in vulnerable communities. This includes causing nutritional deficiencies that lead to chronic cognitive and physical impairments.^2^ More recently, STH infections have been paradoxically linked to both improvement and worsening of inflammatory diseases, such as autoimmune and allergic disorders.^3^^,^^4^ The absence of specific diagnostic tools to identify prior STH exposure has significantly hindered the evaluation of long-term health impact of helminth infections.

The diagnosis of an active STH infection can be accomplished through stool evaluation of helminth eggs using conventional microscopy or by quantitative real-time polymerase chain reaction (qPCR) of helminth DNA in stool.^5^ Although stool-based assays remain essential for research studies and disease control programs, their use in clinical management is limited by cost and required technical expertise, particularly in resource-scarce endemic settings. Another drawback for fecal examinations is that they can only be used to detect active infections and do not provide insight on past exposures.

Serological assays used to identify helminth-specific antibodies could provide a low-cost, user-friendly alternative to detect exposure. Assays using recombinant proteins to detect pathogen-specific host antibodies have a substantial advantage over those relying on crude lysates in their specificity and consistency of results. The improved specificity with a recombinant protein would likely enable better discrimination between STH infections through reduced crossreactivity of host antibodies, an essential feature to any STH diagnostic assay as these parasites coexist in most endemic regions.

Among STHs, serological assays to measure *S. stercoralis* exposure are the only well-validated STH serological assays in humans. Crude antigen immunoassays against *Strongyloides ratti* (Boardier, Switzerland) or* S. stercoralis* (IVD Research, Carlsbad, CA) detect active strongyloidiasis with a sensitivity of 66–91.2% but an inconsistent specificity of 29–100%. Crossreactivity with antibodies generated to other helminth infections, which is common in endemic regions for STHs, restricts the effectiveness of assays using crude antigens in confirming past exposure.^6^^–^^8^ Strongy Detect (InBios, Seattle, WA), a newer serological assay for *S. stercoralis*, minimizes crossreactivity by using helminth-specific recombinant proteins. To develop a recombinant protein-based serological assay for the other STH infections, this study aimed to identify diagnostic candidate STH antigens that could complement the existing stool-based methods used for active infections.

In this study, 236 adolescents and adults living an endemic region of rural Honduras were screened by qPCR of stool, with 56 individuals identified as infected with hookworm, whipworm, and/or roundworm. In addition, sera from 94 age-matched individuals from the United States were included in the study as negative controls. Sera were used to assess the antibody recognition of recombinant antigens specific to two whipworm, four roundworm, and seven hookworm proteins. Most of these recombinant proteins were originally developed as vaccine targets after being identified by immunoscreening of protective sera against crude STH proteins in animal models.^9^

The overall goal of this study was to evaluate the diagnostic potential of 13 STH recombinant proteins. Although one promising candidate, r*Tm*-WAP49, emerged for identifying active whipworm infection, we found discrepancies in serological recognition of previously evaluated STH antigens, highlighting the need to screen multiple endemic populations and consider different platforms before accepting or dismissing a diagnostic candidate.

## MATERIALS AND METHODS

### Honduran study population.

Across two collection periods in 2014 and 2016, we enrolled 113 adolescents (ages 13–17 years old) and 126 adults (ages 18–45 years old) in the Department of Intibucá, Honduras based on known high transmission of STHs in children.^10^ Three participants were subsequently removed for exceeding the age inclusion criteria (over 45 years old) and were reported to the institutional review boards, resulting in a total of 236 participants included in the study.

#### Recruitment, consent, and sample collection.

Before each collection period, local town leaders were engaged by local physician and coauthor C. F. Coello to recruit participants into the study. Persons visiting the Santa Ana Clinic for medical care were not directly recruited into the study. Consenting and sample collection of study participants were held at local community centers, schools, and the Santa Ana Clinic laboratory unit (separate from the medical services), and they were performed by coauthors N. Briggs, G. Segal, S. Novak, P. Lenihan, P. Musgrave, and V. Ellis and overseen by C. F. Coello. Samples were processed and stored appropriately at the laboratory unit of the Santa Ana Clinic until shipped to Baylor College of Medicine for analysis and storage.

#### Inclusion criteria.

Individuals ages 13–45 years old residing in the Department of Intibucá, Honduras who were medically fit to provide stool and blood samples as determined by a local physician were included after obtaining informed consent. Adolescents ages 13–17 years old were required to provide assent in addition to parental or legal guardian consent to participate.

#### Exclusion criteria.

Individuals who had received a deworming medication (albendazole, mebendazole, ivermectin, or pyrantel pamoate) within the past 6 months were excluded from the study. Pregnant women were also excluded. Participants who were not in good health, except those experiencing gastrointestinal symptoms related to a possible parasitic infection as determined by one of our physicians, were excluded and instead, offered medical evaluation by clinic staff separate from this research study.

### U.S. study population.

We established a nonendemic cohort comprising sera from 94 age-matched healthy individuals born in the United States who had no travel history to STH-endemic regions. These samples were collected, processed, and stored in the same manner as the Honduran samples described below.

### Sample processing and preparation.

Approximately 50 g of fresh stool was collected from each subject. DNA was extracted from stool using a modified MP FastDNA for Soil Kit (MP Biochemicals, Santa Ana, CA) as previously described, and the extracted DNA was stored at −80°C.^5^ Serum was obtained through venipuncture, with 2 mL blood collected and immediately centrifuged at 10,000 × *g* for 5 minutes. Approximately 1 mL serum was then stored at −80°C. A positive control for the serological assays was made by pooling equal parts of infected sera for each STH (e.g., sera pool of hookworm-infected persons, etc.). Similarly, a negative sera pool was generated by combining equal parts of sera for all 94 STH-naïve study participants. Sera and stool extracts were stored at −80°C until use.

### Multiparallel qPCR.

DNA primers specific to each STH (*T. trichiura*, *A. lumbricoides*, *A. duodenale*, and *N. americanus*) along with TaqMan MGB probes (Thermo Fisher Scientific, Waltham, MA) were used for the qPCR analyses of DNA extracted from the stool samples to determine STH prevalence and intensity of infection within the Honduran study cohort. The primer sequences and detailed methodology were previously described.^5^

### Antigen preparation.

A total of 13 recombinant proteins for whipworm (r*Tm*-CAP-1 and r*Tm*-WAP49),^11^^,^^12^ roundworm (r*As*14,^13^ r*As*16,^13^ r*As*24,^14^ and r*As*37^15^), and hookworm (r*Na*-SAA-2,^16^ r*Na*-AIP-1,^17^ r*Ac*-TMP-1,^18^ r*Ac*-TMP-2,^19^ r*Ac*16,^20^ r*Na*-GST-1,^21^ and r*Na*-ASP-2^22^) were expressed in *Escherichia coli* or *Pichia pastoris* and purified as previously described.

Crude extracts from dog hookworm *Ancylostoma caninum* L3 and adult worms and from human hookworm *N. americanus* adult worms as well as excretory/secretory (ES) products from whipworm *Trichuris muris* adult worm culture were used as crude antigens and prepared as previously described.^12^^,^^18^^,^^19^ Recombinant proteins and crude lysate were stored at −80°C until use.

### Serological antibody analysis by ELISA.

Repetitive serum samples were evaluated for antigen-specific IgG by indirect ELISA. In brief, 96-well Immunlon 4 HBX plates (Thermo Fisher Scientific, Waltham, MA) were coated with antigen in 1× KPL coating buffer (VWR, Radnor, PA). The ideal coating concentration was established by comparing a range of antigen concentrations from 0.1 to 8 *µ*g/mL and selecting the highest ratio of signal to noise when comparing sera from endemic individuals with known helminth-positive stool with nonendemic control sera. Using the same approach, a standard optimal primary sera concentration across all antigens was established at a 1:400 dilution (range evaluated from 1:100 to 1:800), whereas the secondary anti-human IgG with horseradish peroxidase (HRP) antibody was set at a 1:2,000 dilution (Thermo Fisher Scientific, Waltham, MA). The plate was developed for exactly 5 minutes using KPL SureBlue TMB microwell peroxidase substrate (Thermo Fisher Scientific, Waltham, MA) and stopped using equal parts of 1 M hydrochloric acid. The absorbance was measured by dual-wavelength analysis at test wavelength of optical density (OD) 450 nm minus the reference wavelength of 630 nm using a spectrophotometer (BioTek, Winooski, VT). A set of coated wells that substituted 1× phosphate buffered saline (PBS) for primary sera was used to generate a “blank” well value on every plate. This value was then subtracted from each test well to serve as a control for minor plate-to-plate variations.

We evaluated the 13 recombinant STH proteins mentioned above as serological targets by ELISA. Positive controls for our immunoassays included crude antigens for whipworm (*T. muris* ES) and hookworm (*A. caninum* larval [L3] and adult lysates and *N. americanus* adult lysates). As a positive control for roundworm, we used a total IgG detection kit targeting *A. lumbricoides* crude antigens (Arigio Biolaboratories, Taiwan). In the absence of a gold standard immunoassay to these STHs, we screened candidate antigens using the sera from individuals with corresponding active infection. Thus, sera from 34 individuals with hookworm infection, 23 individuals with ascariasis, and 6 individuals with trichuriasis were used.

Sera from the nonendemic cohort from the United States were split into a screening cohort of 50 randomly selected individuals and the remaining 44 as a validation cohort. After extensive assay optimization for each antigen, the screening cohort generated a 95% cutoff value defined as two SDs of the OD 450 nm from the screening cohort mean. Each cutoff value was then compared between the samples from infected individuals and the validation cohort to determine the sensitivity and specificity. Screening of the entire Honduran (*N* = 236) and U.S. (*N* = 94) populations for IgG recognition of r*Tm*-WAP49 and *Tm*-ES by ELISA was performed in batches because of the large volume of samples. Repeated samples tested between ELISA batches were found to have minor variations. To account for this, replicated negative controls on each plate were averaged and divided by the individual samples on that plate to “normalize” batches. Receiver-operator characteristic (ROC) curves were also generated for each antigen by the Wilson–Brown method in Prism v. 10.3.1.

### Electrophoresis and immunoblotting.

Samples containing recombinant protein or a crude antigen mixture were denatured in lithium dodecyl sulfate buffer supplemented with a reducing agent, dithiothreitol (DTT) or 2-mercaptoethanol (2-ME), unless otherwise indicated, and then, they were boiled for 20 minutes at 98°C. Samples were then separated by 4–12% gradient NuPAGE/Bis-Tris (Invitrogen, Carlsbad, CA) and either stained with Coomassie Blue or transferred onto a polyvinylidene fluoride (PVDF) membrane using the iBlot2 system (Thermo Fisher Scientific). PVDF membranes were blocked with 5% nonfat milk in 1× PBS with 0.05% Tween-20 and then incubated with pooled sera (1:500–1:2,000 for IgG detection and 1:200–1:500 for IgE detection) from *Trichuris*-infected persons or using the pooled naïve cohort. HRP-conjugated goat anti-human IgG or IgE (Thermo Fisher Scientific) at 1:2,000–1:4,000 was used as the secondary antibody as per manufacturer recommendations. Enhanced chemiluminescence (ECL) Prime Western Blotting Detection Reagent (Cytiva, Marlborough, MA) substrate was then used to develop antigen-specific antibody bound bands and visualized on the BioRad ChemiDoc MP with the same exposure time between comparison blots (naïve and infected sera incubated). For the Coomassie stain, 5 *µ*g *Tm*-ES or 2 *µ*g recombinant protein (r*Tm*-WAP49 or r*Tm*-CAP-1) was used. Protein quantity was initially optimized for western blot at 5 *µ*g *Tm*-ES, 2 *µ*g r*Tm*-CAP-1, and 0.25 *µ*g r*Tm*-WAP49.

### Circular dichroism.

Far-ultraviolet (far-UV) circular dichroism (CD) spectra were used to predict the secondary structure of r*Tm*-WAP49 as previously described.^23^ In brief, the CD spectra of r*Tm-*WAP49 were averaged over six scans compared with a baseline of buffer-only sample. The secondary structure was predicted using CDPro software (Colorado State University, For Collins, CO) to compare against reference sets SP43, SDP48, and SMP56 and analyzed using two data-fitting programs, CONTIN and CDSSTR. In addition, CD spectra were collected as a function of temperature to determine the thermodynamics of protein unfolding. The sample was heated from 25°C to 83°C, increasing the temperature stepwise at 1°C per data point. Each data point was determined as the average of two scans.

### High-performance liquid chromatography with fractional collection.

High-performance liquid chromatography (HPLC) was used to collect fractions of the different sizes of r*Tm*-WAP49. The system consisted of an Agilent 1260 Infinity series HPLC coupled with a UV detector (Agilent Technologies, Santa Clara, CA) and a 1260 Infinity II Analytical-Scale Fraction Collector (Agilent Technologies). An aliquot of 200 *µ*g r*Tm*-WAP49 was injected into a TSK gel Super SW2000 column (TOSOH Biosciences, Grove City, OH) and eluted at 0.5 mL/minute isocratically with 1× PBS (pH 7.4) for 35 minutes. After 10 minutes, all fractions were collected in a 96-well plate at approximately 250 *µ*L of volume.

## RESULTS

### High prevalence of STHs among adolescents and adults in Intibucá, Honduras.

**Figure 1.**
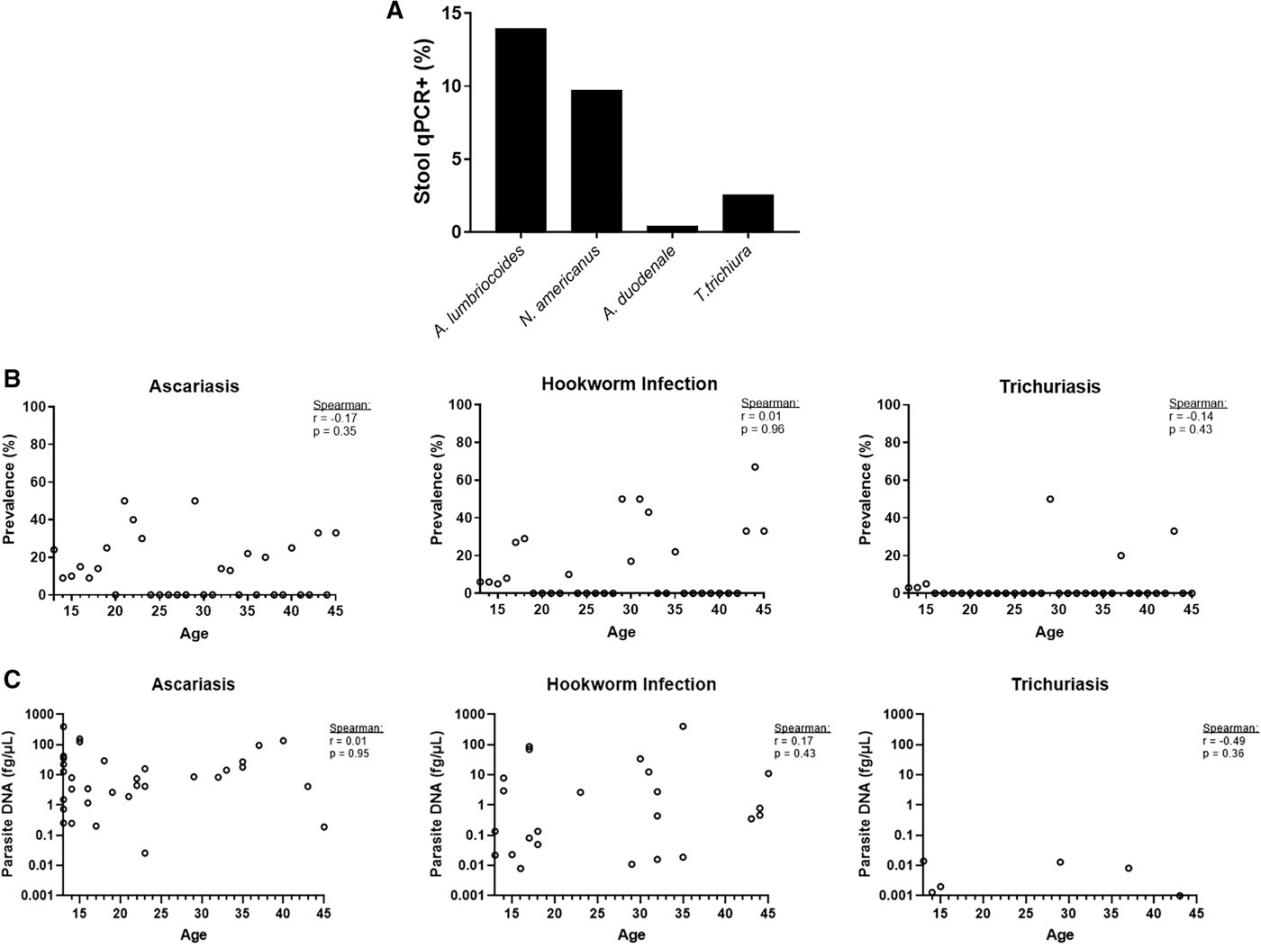
Prevalence and burden of soil-transmitted helminths (STHs) among adolescents and adults in Intibucá, Honduras. STH infections were measured by quantitative real-time polymerase chain reaction (qPCR) amplification of parasite DNA in stool samples. (**A**) Prevalence of roundworm (*Ascaris lumbricoides*), hookworm (*Necator americanus* and *Ancylostoma duodenale*), and whipworm (*Trichuris trichiura*) among the Honduran cohort (*n* = 236). (**B** and **C**) Association of participant age with (**B**) prevalence and (**C**) parasite burden in stool. Correlations were estimated by the calculation of Spearman’s rank correlation coefficients (*r*) and statical significance (*P*-value).

Honduras is a highly endemic nation for STHs, particularly in the rural regions. Although a handful of epidemiological studies have assessed STHs in the Department of Intibucá, Honduras, there are scare available data on the prevalence and disease burden in adolescents and adults.^10^^,^^24^ Among the 236 individuals evaluated in this study, over 50% of otherwise healthy participants exhibited gastrointestinal symptoms at the time of recruitment (Supplemental Table 1). The overall prevalence of STHs was 23.7% (*n* = 56 of 236), including six participants with two or more STH infections. This consisted of 14.0% (*n* = 33 of 236) with *A. lumbricoides*, 0.4% (*n* = 1 of 236) with *A. duodenale*, 9.7% (*n* = 23 of 236) with *N. americanus*, and 2.5% (*n* = 6 of 236) with *T. trichiura* (Figure 1A).

Although global epidemiological studies among adults are limited, studies have suggested that the intensity of STH infections decreases or at least plateaus by adulthood, despite a continued high prevalence, suggesting some degree of acquired immunity.^25^ Yet, in our study, Spearman’s rank-order correlation of age to prevalence (Figure 1B) or burden of infections (Figure 1C) found no relationship of age to either.

### r*Tm*-WAP49 is highly recognized by IgG in the serum of individuals with trichuriasis.

To date, there are no commercially available serological tests to identify active or past infection for hookworm or *Trichuris*, and the few available for *Ascaris* have not been well validated for humans.^26^ Crude parasite lysate was, therefore, used as a control in our in-house IgG ELISA. Hookworm-infected individuals could be diagnosed with 92% sensitivity using *N. americanus* adult lysate in ELISA, and *Trichuris* could be detected with 83% sensitivity. To measure IgG reactivity to *A. lumbricoides* crude lysate, we used a commercially available assay per protocol (Arigio Biolaboratories). After the exclusion of two equivocal results within the cutoff range, there was a sensitivity of 87.5% (*n* = 33) and there were no false-positive results among the five U.S. naïve controls tested. In comparison, among the 13 recombinant protein antigens evaluated, 12 had poor IgG antigen recognition (≤17% sensitivity). However, the *Trichuris* vaccine antigen, r*Tm*-WAP49, was recognized by all six infected individuals (100% sensitivity), and ELISA had few false positives among a nonendemic validation cohort (93% specificity) (Table 1).

**Table 1:** IgG ELISA of soil-transmitted helminth recombinant proteins and crude antigens

Soil-Transmitted Helminth	Protein(s)	Coating Concentration, *µ*g/mL	Sensitivity, %	Specificity, %
*Trichuris*	r*Tm*-CAP-1	0.5	17	93
	r*Tm*-WAP49	0.5	100	91
	*Tm*-ES	0.5	83	93
*Ascaris*	r*As*14	2	3	77
	r*As*16	1	6	95
	r*As*24	2	0	91
	r*As*37	2	3	86
Hookworm	r*Na*-SAA-2	1	4	89
	r*Na*-AIP-1	0.5	13	91
	r*Ac*-TMP-1	2	0	95
	r*Ac*-TMP-2	8	8	91
	r*Ac*16	2	4	95
	r*Na*-GST-1	2	4	93
	r*Na*-ASP-2	1	4	100
	*Ancylostoma caninum* L3 lysate	1	50	100
	*Ancylostoma caninum* adult lysate	1	38	93
	*Necator americanus* adult lysate	1	92	93

The list of the 17 antigens derived from *Trichuris*, *Ascaris*, or hookworm includes 13 recombinant proteins and 4 crude antigen mixtures. An ideal coating concentration of 0.1–8 *µ*g/mL was identified as the highest ratio of signal (sera from whipworm-infected participants) to noise (sera from nonendemic participants) by IgG ELISA. The sensitivity was determined by the number of positive tests (true positives) to negative tests (false negatives) to a particular antigen among infected individuals to the corresponding soil-transmitted helminth infection. The specificity was established as the number of negative tests (true negatives) to positive tests (false positives) among a nonendemic validation cohort (*n* = 44).

**Figure 2.**
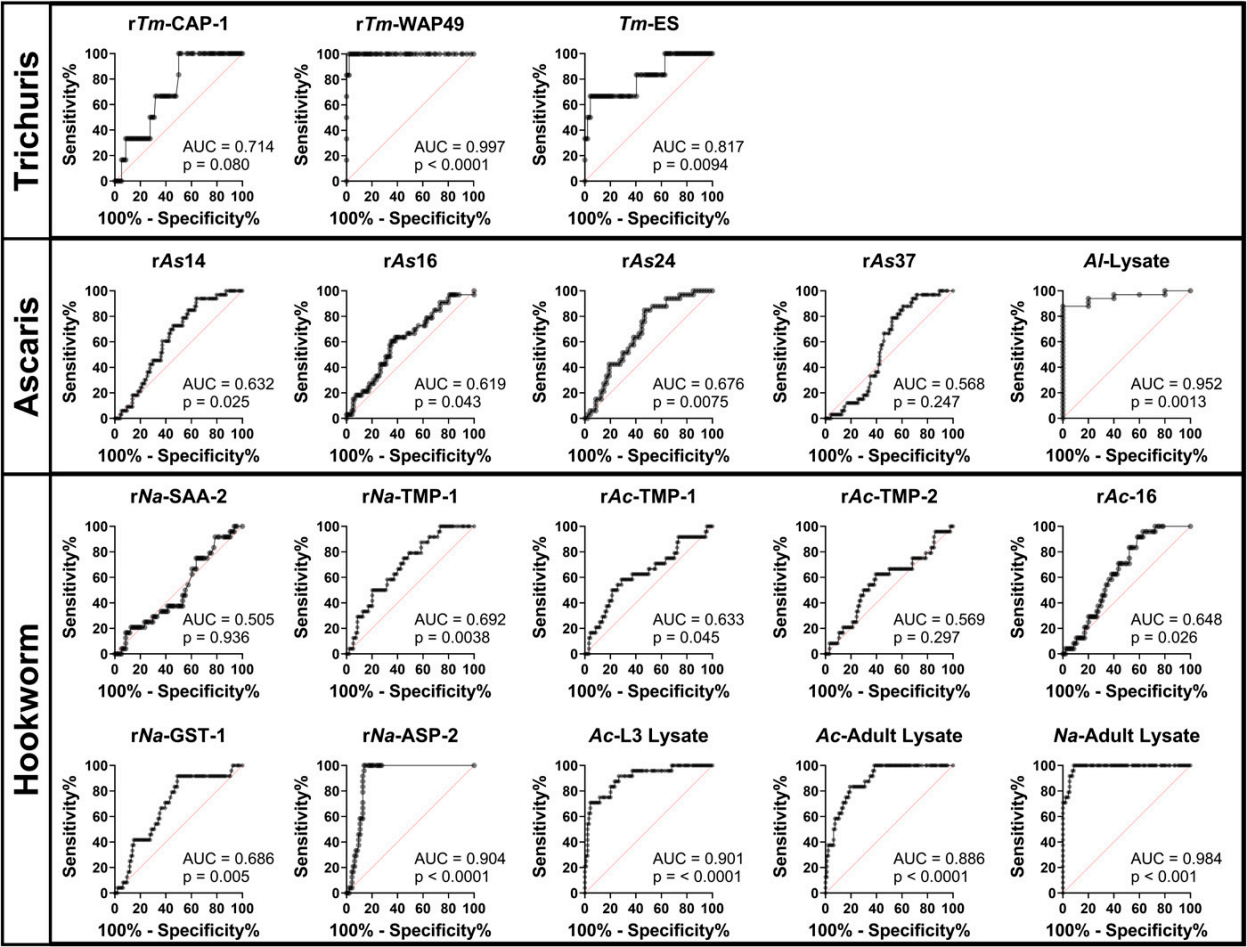
Receiver-operator characteristic (ROC) curves of soil-transmitted helminth recombinant proteins and crude antigen IgG ELISAs. The area under the curve (AUC) for each of the ROC curves is annotated with the 95% CI by the Wilson–Brown method.

An ROC analysis found substantial concordance in the performance characteristics of each antigen to our cutoff methodology (Figure 2). r*Tm*-WAP49 has an area under the curve (AUC) of 0.997 (*P* <0.001), outperforming *T. muris* ES (AUC = 0.817, *P* = 0.0094). The only recombinant protein, otherwise, with an AUC of >0.8 was r*Na*-ASP-2 (AUC = 0.904, *P* <0.0001), comparable with *A. ceylanicum* L3 (AUC = 0.901, *P* <0.0001) and adult (AUC = 0.886, *P* <0.0001) lysates but below *N. americanus* lysate (AUC = 0.984, *P* <0.001).

**Figure 3.**
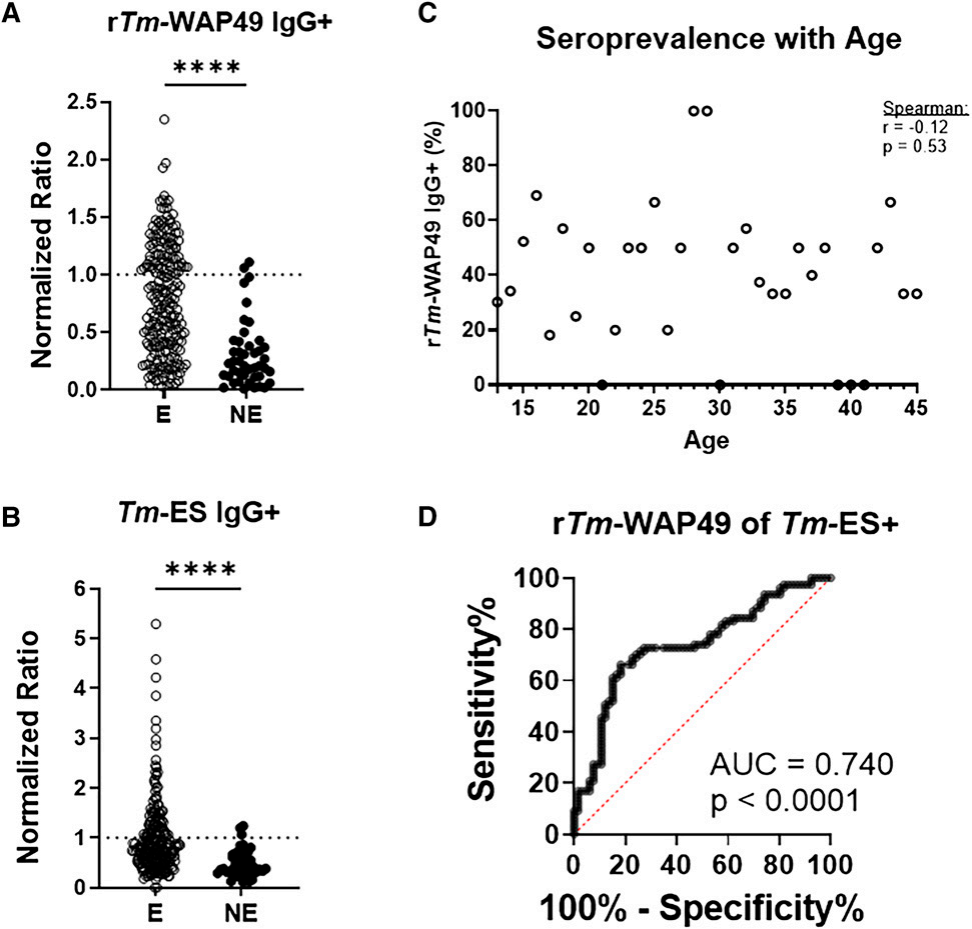
Seroprevalence of r*Tm*-WAP49 and *Tm*-ES. IgG recognition of (**A**) r*Tm*-WAP49 and (**B**) *Tm*-ES as measured by ELISA in the endemic (E) Honduras cohort (*N* = 236) vs. the nonendemic (NE) U.S. cohort (*N* = 94). A 95% positive cutoff was generated in the screening cohort (*n* = 44) on each day that the ELISA testing was performed and normalized to a cutoff of “one.” In the Honduras cohort, 38.1% (*n* = 90 of 236) and 34.3% (*n* = 81 of 236) had IgG recognition of r*Tm*-WAP49 and *Tm-*ES, respectively, compared with 4.5% (*n* = 2 of 44) and 6.8% (*n* = 3 of 44), respectively, in the NE U.S. cohort. (**C**) Serological reactivity to r*Tm*-WAP49 compared with age in the Honduras cohort. Correlations were estimated by calculation of Spearman’s rank correlation coefficients (*r*) and statical significance (*P*-value). (**D**) Receiver-operator characteristic analysis of r*Tm*-WAP49 IgG positivity as a measure of *Tm*-ES IgG seroreactivity in the Honduras cohort (*N* = 236) with an area under the curve (AUC) annotated with the 95% CI by the Wilson–Brown method and statistical significance (*P*-value). ****P <0.0001.

Of the Honduras cohort, 38.1% (*n* = 90 of 236) and 34.3% (*n* = 81 of 236) had IgG recognition of r*Tm*-WAP49 and *Tm-*ES above the screening cohort cutoff, respectively, compared with 4.5% (*n* = 2 of 44) and 6.8% (*n* = 3 of 44), respectively, in the nonendemic U.S. validation cohort (Figure 3A and B). Although we have no means to confirm past or recurrent infections among our participants, there appears to be a persistence of serological reactivity to r*Tm*-WAP49 with age (Figure 3C). An ROC analysis of the concordance of seroreactivity between r*Tm*-WAP49 and *Tm-*ES demonstrated a moderate-strength relationship (AUC = 0.740, *P* <0.0001) (Figure 3D).

**Figure 4.**
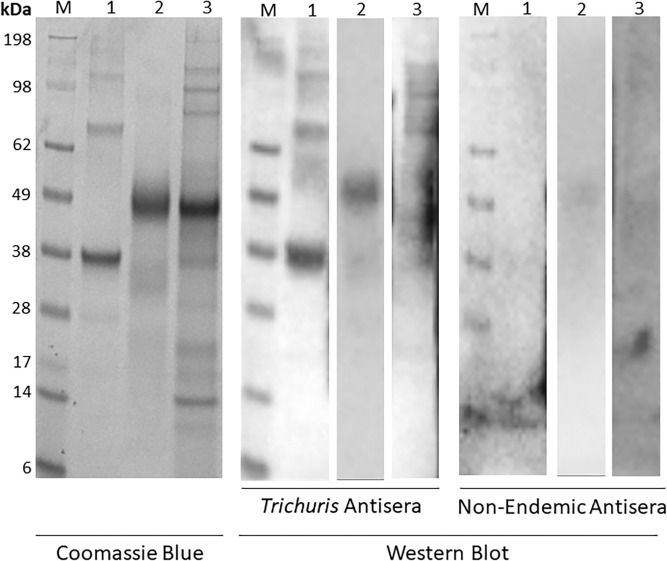
Immunoblotting of *Trichuris* antigens. *Tm*-WAP49 is an immunodominant protein on western blot. Protein separation by 4–12% NuPAGE/Bis-Tris with Coomassie Blue stain (left panel) or transferred to a polyvinylidene fluoride membrane for western blot for protein recognition by pooled sera from *Trichuris*-infected participants (center panel) vs. pooled nonendemic sera (right panel) is shown. For the Coomassie Blue stain, 5 *µ*g *Tm*-ES, 2 *µ*g r*Tm*-CAP-1, and 2 *µ*g r*Tm*-WAP49 were used. For the western blot, 5 *µ*g *Tm*-ES, 2 *µ*g r*Tm*-CAP-1, and 0.25 *µ*g r*Tm*-WAP49 were used. A 1:500 dilution of pooled sera was used for primary incubation of *Tm*-ES and r*Tm*-CAP-1, and a 1:2,000 dilution was used for detection of r*Tm*-WAP49. “M” is the SeeBlue Plus2 prestained protein standard, “1” is r*Tm*-CAP-1 protein, “2” is r*Tm*-WAP49, and “3” is *Tm*-ES.

To further confirm our findings, we pooled sera from individuals infected with whipworm and those from nonendemic regions, and we measured antigen recognition by IgG western blot analyses. A 49-kDa protein corresponding to r*Tm*-WAP49 was detected (Figure 4) at protein concentrations below 0.25 *µ*g and primary sera concentration equal to or above 1:2,000. Importantly, one protein corresponding to the same size as r*Tm*-WAP49 was recognized in crude *Tm*-ES. Although sequencing of this band in *Tm*-ES is necessary to verify the sequence identity, our previous findings of recognition by mouse r*Tm*-WAP49 antisera on western blot analysis further suggest that this band is the native counterpart to r*Tm-*WAP49.^12^ Additionally, r*Tm*-CAP-1 was recognized but only at high protein (2 *µ*g) and primary sera concentrations (1:500). Recognition of r*Tm*-CAP-1 was surprising given that only one individual from the *Trichuris*-infected persons was identified by ELISA. In contrast to the IgG recognition of r*Tm*-WAP49, IgE-specific recognition of r*Tm*-WAP49 required a 10-fold increase in r*Tm*-WAP49 concentration, a 2.5-fold increase in primary sera concentration, and an extended overnight incubation with the primary sera at 4°C (Supplemental Figure 1). We used a similar western blot analyses approach to validate our ELISA findings for the hookworm and roundworm recombinant proteins but did not observe a difference in recognition by the pooled infected over nonendemic sera (data not shown).

### Cysteine-rich r*Tm-*WAP49 exhibits remarkable thermostability and oligomerizes through disulfide bonds.

Based on our data showing WAP49 (WormBase accession code TMUE_s0165000300) to be a potential diagnostic candidate in addition to its known relevance for vaccine development,^11^^,^^12^ we evaluated the biophysical characteristics of r*Tm*-WAP49. The far-UV CD analyses revealed r*Tm*-WAP49 to be composed of approximately 65% turns and loops, 30% β-pleated sheets, and 5% α-helices (Supplemental Figure 2A). To determine the thermal stability and denaturation characteristics, the molar ellipticity at 208 nm of r*Tm*-WAP49 was measured over an increasing temperature range from 25°C to 83°C. A melting temperature of 75°C was identified with a sharp denaturation (Supplemental Figure 2B), suggesting a well-defined low-energy state that keeps the protein folded.

When denatured and reduced through DTT or 2-ME treatment, r*Tm*-WAP49 appears as a single heavily smeared band around 49 kDa on a sodium dodecyl sulfate polyacrylamide gel electrophoresis (SDS-PAGE) gel stain. In the absence of reducing agents, r*Tm*-WAP49 shows three distinct bands (Supplemental Figure 2C). To verify that these bands were not a result of protein instability, we used size-exclusion HPLC to collect the nonreduced fractions. Three distinct fractions were isolated corresponding to 49, 96, and 147 kDa (Supplemental Figure 2D), consistent with observations from the nonreduced SDS-PAGE analysis. These peaks were found to be solitary and stable, suggesting that these three sizes correspond to oligomeric states of r*Tm*-WAP49 as monomer, dimer, and trimer. Given the stability of these fractions to remain in the same oligomeric states only under nonreducing conditions, it appears that these oligomeric states are more likely from disulfide binding between monomeric proteins during early protein folding.

## DISCUSSION

STHs infect nearly a quarter of the world’s population. Yet, there are no well-validated serological tools to aid in their diagnosis or epidemiological examination of past exposure or to study their association with the development of noncommunicable disease conditions, such as autoimmunity, allergy, and cancer.^26^ Helminth crude antigens have been used in isolated research studies, but crossreactivity to other helminth exposures in nearly universal coendemic STH regions further complicates these studies. In this study, we evaluated 13 recombinant STH proteins as serological diagnostic candidates among STH-infected individuals from Honduras.

The recombinant proteins tested in this study were originally developed for vaccine consideration because of their immunodominant association with protection against challenge in animal models. After extensive ELISA optimization, only one promising candidate emerged, r*Tm*-WAP49, for diagnosis trichuriasis in humans. Recognition of this recombinant protein shows excellent overlap in recognition with crude antigen *Tm*-ES, with improved sensitivity. A limitation of our study was that of 236 adolescents and adults, only 6 had active trichuriasis. When testing was expanded to include individuals not infected with trichuriasis, we found that more than one in three persons had antibody-specific recognition. A larger validation study of r*Tm*-WAP49 in a geographical distinct endemic population is underway. Interestingly, in a recent trial of r*Tm*-WAP (the same protein as r*Tm-*WAP49) in an endemic Mozambique population, Luminex multiplex used to measure antigen-specific IgG was highly reactive in both endemic and nonendemic samples but could not discriminate well among those actively infected.^27^ This may be because of the inherent reactivity of the protein to antibodies, which we saw at higher protein concentrations with ELISA and immunoblotting. Thus, refined optimization may be key or simply, a different assay approach for this protein. There may also be batch variations from different fermentations as we suspect that our recombinant protein and the native protein are heavily glycosylated. However, we observed reproducible ELISA results with our two available batches.

Despite strong recognition by crude STH antigens (adult *N. americanus*, adult and L3 *A. duodenale*, adult *A. lumbricoides*, and adult *T. muris* ES), the remaining 12 recombinant protein candidates had poor sensitivity and are unlikely to be of diagnostic value for a serological test. Recombinant *Na*-ASP-2, which was widely reactive by ELISA IgG testing of children and adults in a hookworm-endemic region of Minas Gerais, Brazil,^28^ was also found to have the widest AUC by ROC analysis but poor recognition (4%) by our 95% cutoff strategy. This discrepancy may be because of a relatively low signal-to-noise ratio, which may be an inherent flaw of the antigen as a diagnostic candidate without further optimization As expected, we did not see any serological recognition against r*Na*-GST-1, confirming data collected in Brazil ahead of its use in a phase 1 vaccine clinical trial.^29^ Given the possible discrepancies in r*Tm*-WAP49 recognition in the Mozambique study, we will need to validate our findings in a geographical distinct cohort before concluding the antigen’s full diagnostic potential. Previously, Palmer et al.^30^ found that IgG4 anti-*Necator* antibody showed some association with both prevalence and intensity; therefore, future testing should include a comparison of subclass antibodies.

Crossreactivity from other active or prior helminth infections remains unknown and will be an important future diagnostic consideration. Sera from human STH challenge models or animal models immunized with human STH lysates (such as *N. americanus* adult lysate) and evaluation for crossreactivity to diagnostic candidates for other helminths, like r*Tm*-WAP49, may be a more feasible way to screen because STHs are largely coendemic.

The prevalence of STH infections among adolescents and adults in our study is consistent to what is known for younger children (younger than 13 years old) in this region of Honduras.^10^ Although the prevalence of infection is known to peak in early childhood and remain stable with age, the burden of STH infection is thought to plateau and even decline in adulthood, suggesting acquired immunity.^9^^,^^25^ Although the prevalence of STH infection was steady among our cohort, as expected, the burden of infection did not change with age. Because this study did not evaluate preadolescents or follow participants longitudinally, it is possible that the peak intensity of infection occurs at ages much younger than currently suggested or that acquired immunity occurs in only some individuals. Recent studies have also identified adults as a critical reservoir population of STHs amidst global mass drug administration efforts.^31^ Further studies are needed to address the question of acquired immunity to STHs in endemic regions.

An ideal field test for serological detection of STHs would be a low-cost assay requiring minimal training with easy portability and stability in a range of conditions. r*Tm*-WAP49 has several biophysical characteristics that make it a promising diagnostic candidate. For starters, we have found that it is not difficult to produce the recombinant protein in large quantities by yeast fermentation, and it has conserved serorecognition between batches in our study.^12^ The cysteine-rich protein contains several 50 amino repeats, leading to remarkable thermostability, with a melting temperature of 75°C; thus, the protein may be more resilient to degradation in different storage or testing conditions. Serological recognition is maintained whether the protein is in its native form of oligomers as shown by ELISA or when it is reduced and denatured as seen by western blot. An additional feature of an ideal serological assay would be the ability to distinguish active versus past STH infection. Our data suggest that exposure to r*Tm*-WAP49 leads to persistent antibodies, and thus, identifying a second diagnostic target that could discriminate active infection should be a long-term goal.

In summary, after screening 13 recombinant proteins for antibody recognition among an STH endemic Honduran cohort, we found 1 recombinant protein, r*Tm*-WAP49, with excellent sensitivity and specificity. A larger validation study of r*Tm*-WAP49 in a geographically distinct endemic population is underway.

## Supplemental Materials

10.4269/ajtmh.24-0514 Supplemental Materials
