# Supplementary material for: A Honduran Prevalence Study on Soil-Transmitted Helminths Highlights Serological Antibodies to Tm-WAP49 as a Diagnostic Marker for Exposure to Human Trichuriasis
Source: Am J Trop Med Hyg. 2025 Feb 11;112(5):1017–25. doi: 10.4269/ajtmh.24-0514 (PMC12062693; doi:10.4269/ajtmh.24-0514)
Supplement: Supplemental Materials [file tpmd240514.SD1.pdf]

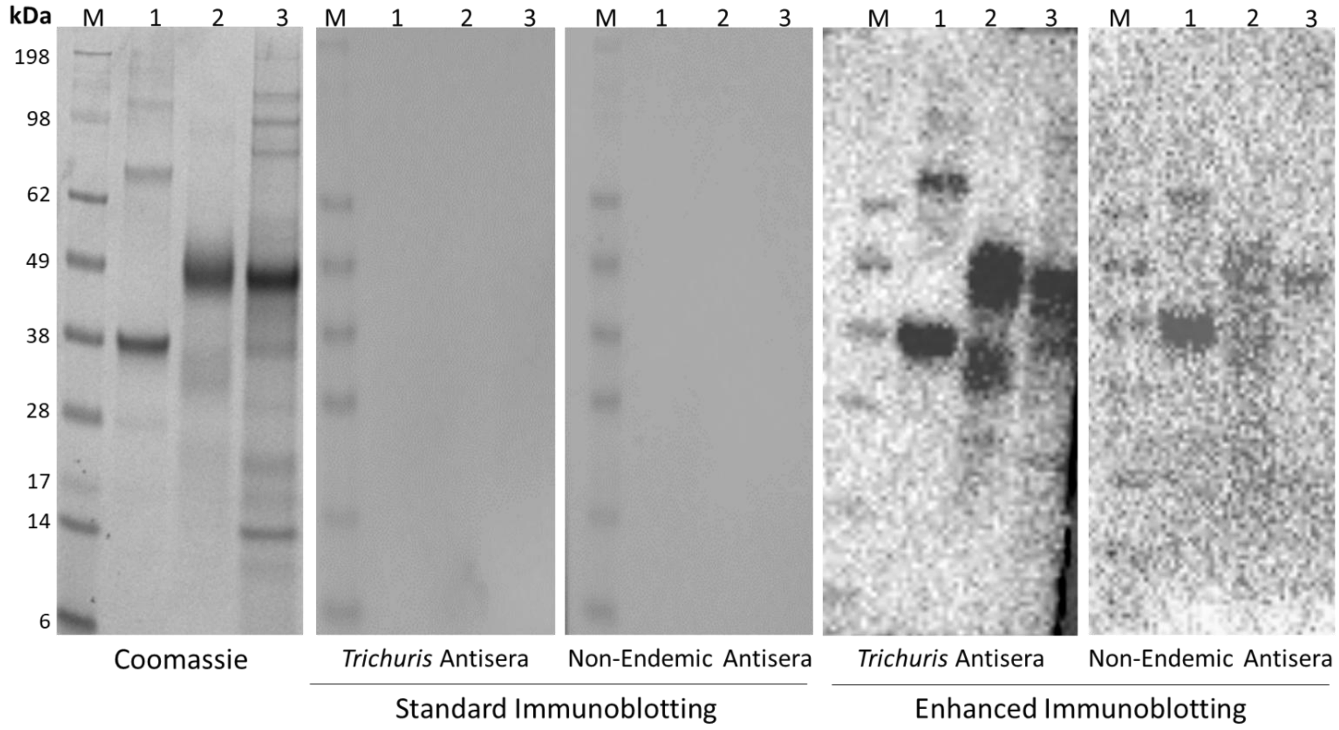

Supplemental Figure 1: Low IgE recognition of r*Tm*-WAP49 on immunoblotting. Protein separation by 4-12% NuPAGE/Bis-Tris with Coomassie stain or transferred to PVDF membrane for chemiluminescent western blot for protein recognition by pooled sera from *Trichuris*-infected Hondurans versus pooled nonendemic sera followed by secondary anti-human IgE coupled with HRP. Protein recognition was compared using a standardized (0.25 µg of recombinant protein or 5 µg of *Tm*-ES, 1:500 dilution of primary sera, and primary sera incubated at room temperature for 1 hour) versus enhanced protocol (2 µg of protein or 5 µg of *Tm*-ES, 1:200 dilution of primary sera, and primary sera incubated at 4°C overnight). For the Coomassie stain, 5 µg of *Tm*-ES or 2 µg of recombinant protein (r*Tm*-WAP49 or r*Tm*-CAP-1) was used. “M” is the SeeBlue Plus2 pre-stained protein standard, “1” is r*Tm*-CAP-1 protein, “2” is r*Tm*-WAP49, and “3” is *Tm*-ES.

A

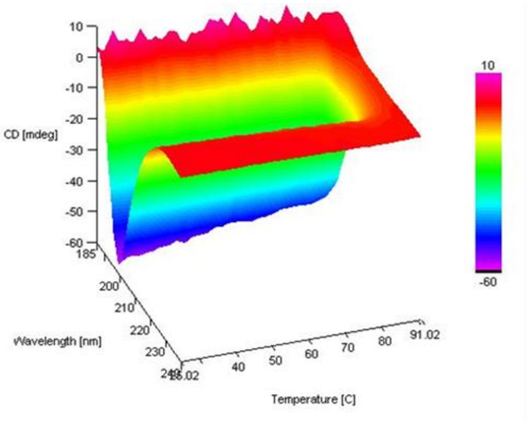

Predicted protein structure(%)		
	CD data	Phyre 2 model
Helix	5	6
Beta sheet	30	0
Turns and loops	65	94

B

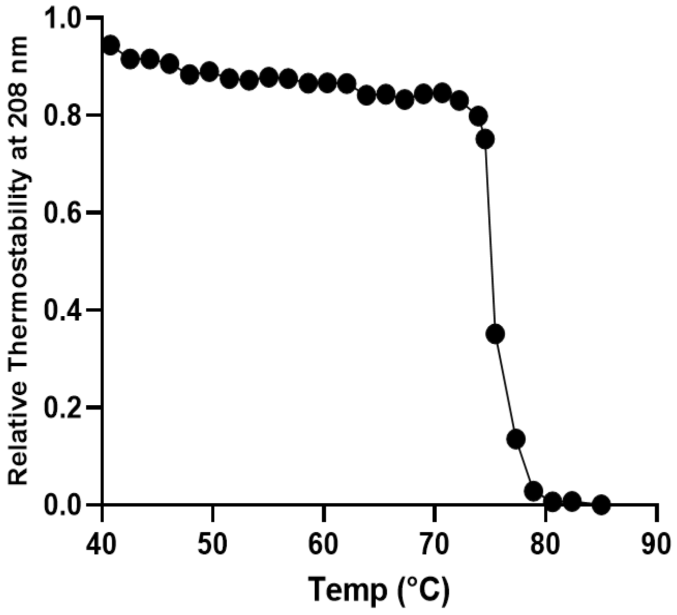

C

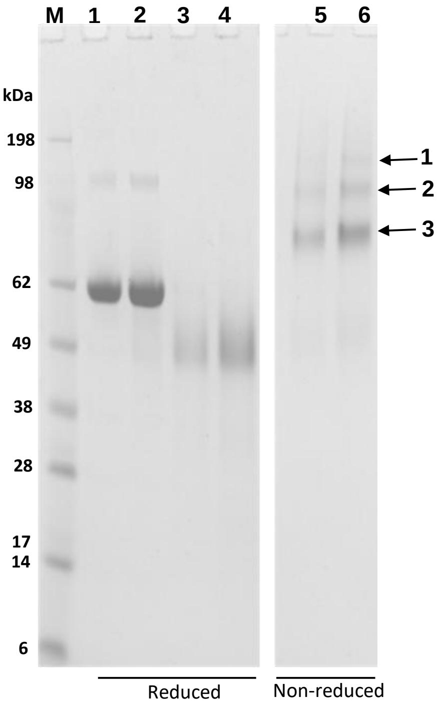

D

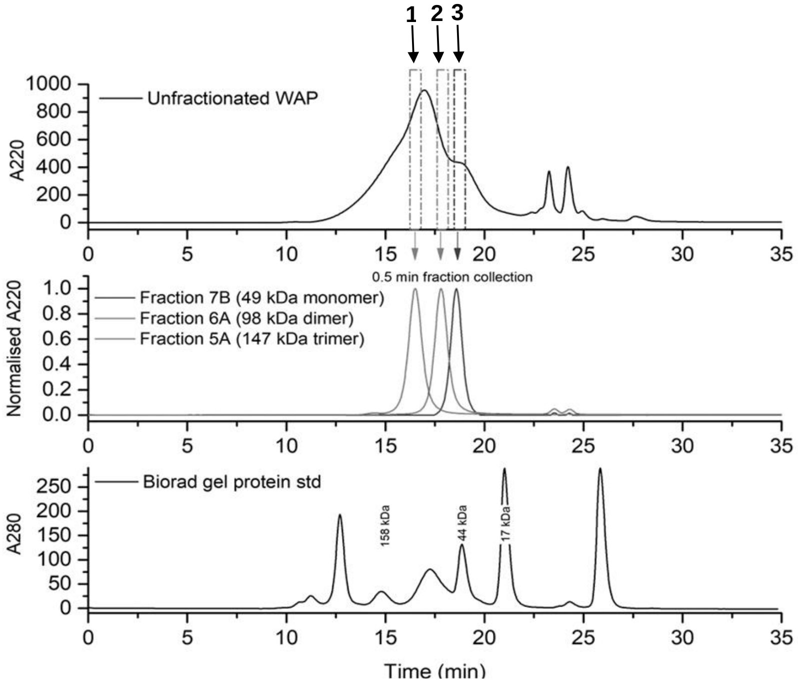

Supplemental Figure 2. Disulfide bonds of cysteine-rich r*Tm*-WAP49 facilitates high thermostability and oligomerization. (A) Circular dichroism (CD) spectra were scanned from 190 nm to 300 nm at constant temperature (25°C) with predicted secondary structure grouped into the categories of helices, beta sheets, and turns and loops. (B) The thermodynamics of r*Tm*-WAP49 was determined by heating the protein in stepwise 1°C/data point from 25°C to 83°C and measuring the relative CD thermostability at a molar ellipticity of 208 nm. (C) Comparison of reduced vs non-reduced r*Tm*-WAP49 on SDS-PAGE: (M) SeeBlue pre-stained protein marker; (1-2) BSA control at 1 µg and 2 µg; (3-4) reduced r*Tm*-WAP49 at 1 µg and 2 µg; and (5-6) non-reduced r*Tm*-WAP49 at 1 µg and 2 µg. The SDS-PAGE was performed using 4-20% NuPAGE/MES gel and stained with Coomassie Brilliant Blue. (D) 2 µg of *rTm*-WAP49 was injected into a TSK gel Super SW2000 column and eluted at 0.3 ml/min isocratically with PBS (pH 7.4) for 9 min. Absorbance was continuously measured at 220 nm. Molecular weight standard (MWS) was used (bottom figure) and peaks a, b, c, d, and e, correspond with respective MW of 670 kDa, 158 kDa, 44 kDa, 17kDa and 1.35 kDa. Adapted with permission from TMC Digital Commons.[29]

Baseline characteristics

Characteristic	Callejones (n = 6)	Colomoncagua centro (n = 92)	El Carrizal (n = 17)	El Hondable (n = 8)	Llano Grande (n = 11)	San Antonio Vados (n = 20)	Santa Ana (n = 82)	Total (n = 236)
**Sex**								
Male	1	39	6	5	5	7	26	89
Female	5	53	11	3	6	13	56	147
**Age** (years, mean)	14.0	19.3	25.6	21.6	22.1	22.1	25.9	23.2
**G.I. symptoms**								
G.I. pain (%)	50%	38%	30%	60%	43%	60%	28%	44%
Diarrhea (%)	33%	10%	20%	20%	0%	15%	3%	14%
Constipation (%)	17%	7%	10%	20%	0%	10%	3%	9%
**Prevalence**								
A. lumbricoides (%)	16.7%	13.0%	11.8%	12.5%	0.0%	30.0%	13.4%	14.0%
T. trichiura (%)	0.0%	4.3%	0.0%	0.0%	0.0%	0.0%	2.4%	2.5%
N. americanus (%)	16.7%	4.3%	0.0%	12.5%	18.2%	30.0%	12.2%	9.7%
A. duodenale (%)	0.0%	0.0%	0.0%	0.0%	0.0%	0.0%	1.2%	0.4%
Any STH Infection (%)	33%	20%	12%	25%	18%	50%	26%	23.7%

Supplemental Table 1: Baseline characteristic and STH prevalence by stool qPCR of participants in seven villages in Intibucá, Honduras. Participants were surveyed for age, sex, and gastrointestinal (G.I.) symptoms (pain, diarrhea, constipation).
